# Supplementary material for: Comparison of hospitalization events among residents of assisted living and nursing homes during COVID-19: Do settings respond differently during public health crises?
Source: PLoS One. 2024 Jul 12;19(7):e0306569. doi: 10.1371/journal.pone.0306569 (PMC11244779; doi:10.1371/journal.pone.0306569)
Supplement: S1 Table — (DOCX) [file pone.0306569.s001.docx]

**S1 Table. Description of Alberta Provincial Clinical and Health Administrative Databases.**

| **DATABASE** | **DESCRIPTION** |
| --- | --- |
| Discharge Abstract Database (DAD) | Contains patient-level data (demographic, diagnoses, procedures, relevant dates) for all admissions to acute care hospitals in Alberta.  Data available from Apr 1, 2002. |
| Vital Statistics | Contains dates of death for residents of Alberta.  Data available from Jan 1, 1999. |
| Alberta Continuing Care Information System (ACCIS) Database | Contains data for all continuing care (Designated Supportive Living [DSL] and Long-Term Care [LTC]) residents and homes in Alberta.  Includes all mandatory clinical assessments (Resident Assessment Instrument – Home Care [RAI-HC] and Resident Assessment Instrument – Minimum Data Set 2.0 [RAI-MDS 2.0) administered among DSL and LTC residents, respectively, in Alberta. These standardized assessments are performed by trained healthcare personnel and collect information on a wide range of health indices including cognitive function, performance on activities of daily living, mood and behaviours, health instability, psychosocial well-being, and clinical diagnoses.  Also available are measures of DSL/LTC home bed size, ownership status, rural/urban residence, and health zone location in the province (the latter two characteristics based on home postal code).  Data available from Jan 1, 2010 onward. |
| Provincial Laboratory COVID-19 testing data | Contains information on COVID-19 infections (date of test, results), including testing for all DSL and LTC residents in Alberta. COVID-19 data available from Feb 1, 2020 onward. |
| Immunization and Adverse Reactions to Immunization (ImmARI) | Contains information on vaccines administered including date and type of vaccination. Includes vaccines administered in various settings such as public health clinics, pharmacies, and physician offices. Data were used with approval from Alberta Health (data custodian). |
