# Supplementary material for: Comparison of hospitalization events among residents of assisted living and nursing homes during COVID-19: Do settings respond differently during public health crises?
Source: PLoS One. 2024 Jul 12;19(7):e0306569. doi: 10.1371/journal.pone.0306569 (PMC11244779; doi:10.1371/journal.pone.0306569)
Supplement: S2 Table — (DOCX) [file pone.0306569.s002.docx]

**S2 Table. Adjusted rate ratios^a^ for all-cause hospitalization, hospitalization with delayed discharge, and hospitalization with death, comparing COVID-19 pandemic vs historical (2018/19) monthly periods, among Alberta Assisted Living (AL) and Nursing Home (NH) residents.**

|  | **Rate Ratio (95% CI) for Hospital Outcome Associated with Select COVID-19 Pandemic Time Periods** | | | | | |
| --- | --- | --- | --- | --- | --- | --- |
| **Hospital Outcome,**  **Setting** | April 2020  [Peak Wave 1] | August 2020 | December 2020  [Peak Wave 2] | January 2021 | April 2021  [Peak Wave 3] | September 2021  [Peak Wave 4] |
| ***All-Cause Hospitalization*** |  |  |  |  |  |  |
| Period (pandemic vs 2018/19)  AL  NH | **0.60 (0.51-0.71)**  **0.74 (0.64-0.85)** | **0.87 (0.76-1.00)**  **0.82 (0.71-0.95)** | 0.92 (0.80-1.04)  **0.82 (0.72-0.95)** | 1.05 (0.92-1.18)^c^  **0.82 (0.72-0.95)^c^** | **0.84 (0.73-0.97)**  **0.85 (0.74-0.98)** | **0.76 (0.66-0.88)**  **0.65 (0.56-0.75)** |
| Setting (AL vs NH)  Pandemic  2018/19 | **1.25 (1.02-1.53)**  **1.53 (1.33-1.76)** | **1.58 (1.32-1.89)**  **1.49 (1.30-1.71)** | **1.80 (1.51-2.15)**  **1.62 (1.42-1.85)** | **2.03 (1.71-2.40)**  **1.60 (1.40-1.82)** | **1.47 (1.22-1.78)**  **1.48 (1.29-1.71)** | **1.64 (1.34-2.00)**  **1.40 (1.22-1.60)** |
| ***Hospitalization with Delayed Discharge^b^*** |  |  |  |  |  |  |
| Period (pandemic vs 2018/19)  AL  NH | 0.98 (0.71-1.34)^d^  **1.94 (1.31-2.85)^d^** | 1.14 (0.87-1.51)  **1.84 (1.19-2.86)** | 0.98 (0.73-1.33)  1.39 (0.89-2.15) | 1.16 (0.88-1.52)  1.15 (0.73-1.80) | 0.81 (0.57-1.15)  0.82 (0.49-1.38) | 0.71 (0.50-1.01)  0.63 (0.35-1.15) |
| Setting (AL vs NH)  Pandemic  2018/19 | **2.32 (1.52-3.54)**  **4.60 (3.11-6.81)** | **4.03 (2.61-6.22)**  **6.49 (4.19-10.06)** | **3.88 (2.41-6.24)**  **5.47 (3.66-8.16)** | **4.51 (2.87-7.09)**  **4.47 (3.02-6.62)** | **4.45 (2.50-7.93)**  **4.53 (3.00-6.85)** | **5.38 (2.78-10.40)**  **4.81 (3.11-7.44)** |
| ***Hospitalization with Death*** |  |  |  |  |  |  |
| Period (pandemic vs 2018/19)  AL  NH | 0.80 (0.55-1.17)  1.05 (0.75-1.46) | 1.27 (0.88-1.82)  0.81 (0.57-1.14) | **1.89 (1.40-2.54)**  **1.53 (1.14-2.05)** | **1.47 (1.07-2.01)**  0.99 (0.72-1.37) | **0.50 (0.31-0.79)**  0.71 (0.48-1.05) | 1.20 (0.77-1.87)  0.90 (0.63-1.29) |
| Setting (AL vs NH)  Pandemic  2018/19 | 1.27 (0.81-2.00)  **1.66 (1.19-2.32)** | **2.37 (1.52-3.71)**  **1.51 (1.07-2.12)** | **2.13 (1.50-3.01)**  **1.73 (1.24-2.41)** | **2.13 (1.43-3.17)**  1.44 (0.72-1.37) | 1.05 (0.58-1.89)  **1.50 (1.06-2.12)** | 1.40 (0.84-2.34)  1.05 (0.70-1.58) |

a For each time period, separate Poisson regression generalized estimating equations (GEE) models were used to estimate rate ratios for period (COVID-19 pandemic vs historical [2018/19]), setting (AL vs NH) and period-setting interactions; Models adjusted for age, sex, ADL, CPS, CHESS, #chronic conditions, health zone & ownership status.

b Note: An adjusted model for October 2020 was also computed given the rise in rate of hospitalization with a delayed discharge evident in Figures 2 and S2: for AL the adjusted RR=1.55 (1.17-2.04), for NH the adjusted RR=2.57 (1.69-3.91), with a statistically significant interaction (period*setting), p=0.048

c Test of statistical significance for interaction of period*setting, p=0.013

d Test of statistical significance for interaction of period*setting, p=0.008
