# Supplementary material for: Comparison of hospitalization events among residents of assisted living and nursing homes during COVID-19: Do settings respond differently during public health crises?
Source: PLoS One. 2024 Jul 12;19(7):e0306569. doi: 10.1371/journal.pone.0306569 (PMC11244779; doi:10.1371/journal.pone.0306569)
Supplement: S3 Table — B) Distribution of top 10 ICD-10-CA diagnosis / chapters for most responsible diagnosis associated with hospitalizations during April 2020 and January 2021 (relative to comparable historical 2018/19 months), by setting. (DOCX) [file pone.0306569.s003.docx]

**S3 Table.**

1. **Distribution of top 10 ICD-10-CA diagnosis (grouped) for most responsible diagnosis associated with hospitalizations during April 2020**

**and January 2021 (relative to comparable historical 2018/19 months), by setting.**

| **April 1, 2020 [Peak Wave 1]** | | **April 1, 2018-2019** | |
| --- | --- | --- | --- |
| **AL** | **NH** | **AL** | **NH** |
| 14.1 Injury, Fracture | 12.4 Respiratory disease | 17.6 Respiratory disease | 19.9 Respiratory disease |
| 10.1 Respiratory disease | 11.2 Renal, UTI | 6.7 Circulatory disease | 8.7 Renal, UTI |
| 8.3 Dementia, Mental health, Delirium | 9.6 Injury, Fracture | 6.2 Renal, UTI | 6.0 Injury, Fracture |
| 5.4 Circulatory disease | 6.8 Sepsis, Bacterial infection | 5.7 Injury, Fracture | 5.4 Circulatory disease |
| 5.3 Renal, UTI | 4.8 COVID-19 | 4.5 Dementia, Mental health, Delirium | 4.1 Palliative care |
| 3.9 Palliative care | 4.4 Circulatory disease | 4.4 Palliative care | 2.9 Sepsis, Bacterial infection |
| 1.5 Sepsis, Bacterial infection | 4.4 Palliative care | 2.1 Abnormal signs/symptoms NOS | 1.7 Abnormal signs/symptoms NOS |
| 1.5 Cellulitis | 3.6 Dementia, Mental health, Delirium | 1.0 Gastrointestinal condition | 1.5 Dementia, Mental health, Delirium |
| 1.0 Hyperosmolality, Hypernatraemia | 1.2 Gastrointestinal condition | 1.0 Cellulitis | 1.4 Cellulitis |
|  | 0.8 Hyperosmolality, Hypernatraemia | 0.8 Sepsis, Bacterial infection | 0.9 Hyperosmolality, Hypernatraemia |

| **January 1, 2021 [Noted difference between settings]** | | **January 1, 2018-2019** | |
| --- | --- | --- | --- |
| **AL** | **NH** | **AL** | **NH** |
| 15.0 COVID-19 | 11.1 COVID-19 | 17.0 Respiratory disease | 20.9 Respiratory disease |
| 9.7 Respiratory disease | 9.7 Respiratory disease | 7.8 Renal, UTI | 6.5 Renal, UTI |
| 9.3 Injury, Fracture | 8.7 Renal, UTI | 6.8 Injury, Fracture | 5.4 Injury, Fracture |
| 6.1 Dementia, Mental health, Delirium | 6.2 Injury, Fracture | 6.5 Circulatory disease | 4.4 Circulatory |
| 5.8 Circulatory disease | 4.5 Palliative care | 4.7 Dementia, Mental health, Delirium | 4.4 Palliative care |
| 4.7 Renal, UTI | 4.5 Sepsis, Bacterial infection | 2.8 Palliative care | 3.5 Dementia, Mental health, Delirium |
| 3.7 Palliative care | 3.5 Circulatory disease | 1.1 Cellulitis | 2.8 Gastrointestinal condition |
| 2.0 Abnormal signs/symptoms NOS | 2.1 Hyperosmolality, Hypernatraemia | 1.0 Sepsis, Bacterial infection | 1.7 Sepsis, Bacterial infection |
| 1.0 Gastrointestinal condition | 1.7 Gastrointestinal condition | 1.0 Abnormal signs/symptoms NOS | 1.2 Cellulitis |
| 0.8 Sepsis, Bacterial infection | 1.0 Dementia, Mental health, Delirium |  | 1.0 Hyperosmolality, Hypernatraemia |
|  | 1.0 Idiopathic thrombocytopenic purpura |  |  |

1. **Distribution of top 10 ICD-10-CA diagnosis / chapters for most responsible diagnosis associated with hospitalizations during April 2020**

**and January 2021 (relative to comparable historical 2018/19 months), by setting.**

|  | | **April 1, 2020** | | **April 1, 2018-2019** | | **Jan 1, 2021** | | **Jan 1, 2018-2019** | |
| --- | --- | --- | --- | --- | --- | --- | --- | --- | --- |
| **Total cohort size on monthly index date** | | **AL (9,565)** | **NH (14,410)** | **AL (17,506)** | **NH (27,994)** | **AL (9,312)** | **NH (13,511)** | **AL (17,374)** | **NH (27,895)** |
| **# Inpatient hospitalizations** | | 206 | 251 | 630 | 667 | 401 | 289 | 728 | 729 |
| **#ALC bed stay** | | 62 | 52 | 110 | 53 | 86 | 30 | 136 | 57 |
| **Median (IQR) ALC bed days for those with ALC stay** | | 24  (13-56) | 13  (6.5-24) | 28  (12-52) | 9  (3-25) | 30  (6-52) | 9.5  (4-28) | 17  (7-42) | 8  (4-14) |
| **A00-B99-Certain infectious & parasitic diseases (%)** | |  |  |  |  |  |  |  |  |
| A41.9 | Sepsis, unspec. | 1.5 | 3.6 | 0.8 | 2.1 | 0.8 | 2.8 | 1.0 | 1.7 |
| A41.50 | Sepsis due to E.coli |  | 2.4 |  | 0.8 |  | 1.0 |  |  |
| A41.58 | Sepsis due to other gram-negative organisms |  | 0.8 |  |  |  |  |  |  |
| A49.0 | Staphylococcal infection, unspec site |  |  |  |  |  | 0.7 |  |  |
|  | **[Total]** | **[1.5]** | **[6.8]** | **[0.8]** | **[2.9]** | **[0.8]** | **[4.5]** | **[1.0]** | **[1.7]** |
| **D50-D89-Diseases of the blood/blood-forming organs & certain disorders involving immune system (%)** | |  |  |  |  |  |  |  |  |
| D69.38 | Other idiopathic thrombocytopenic purpura |  |  |  |  |  | 1.0 |  |  |
| **E00-E90-Endocrine, nutritional & metabolic diseases (%)** | |  |  |  |  |  |  |  |  |
| E87.0 | Hyperosmolality & hypernatraemia | 1.0 | 0.8 |  | 0.9 |  | 2.1 |  | 1.0 |
|  | |  |  |  |  |  |  |  |  |
| **F00-F99-Mental & behavioural disorders (%)** | |  |  |  |  |  |  |  |  |
| F01.9 | Vascular dementia, unspec. | 1.0 | 0.8 |  |  |  |  |  |  |
| F03 | Unspec. dementia | 3.9 | 2.0 | 2.2 | 1.5 | 4.5 |  | 2.3 | 1.4 |
| F05.1 | Delirium superimposed on dementia | 1.9 |  | 1.3 |  |  |  | 1.2 | 1.1 |
| F05.8 | Other delirium |  |  |  |  | 0.8 |  |  | 1.0 |
| F05.9 | Delirium unspec |  |  | 1.0 |  |  |  | 1.2 |  |
| F25.9 | Schizoaffective disorder, unspec |  | 0.8 |  |  |  |  |  |  |
| **G00-G99-Diseases of the nervous system (%)** | |  |  |  |  |  |  |  |  |
| G20 | Parkinson’s disease |  |  |  |  | 0.8 | 1.0 |  |  |
| G30.9 | Alzheimer disease, unspecified | 1.5 |  |  |  |  |  |  |  |
|  | **[Total]** | **[8.3]** | **[3.6]** | **[4.5]** | **[1.5]** | **[6.1]** | **[1.0]** | **[4.7]** | **[3.5]** |
| **I00-I99-Diseases of circulatory system** | |  |  |  |  |  |  |  |  |
| I21.4 | Acute subendocardial MI | 1.5 | 0.8 | 1.1 | 1.2 |  |  | 1.0 |  |
| I26.9 | Pulmonary embolism without mention of acute cor pulmonale |  |  | 1.0 |  |  |  |  |  |
| I48.90 | Afib, unspecified |  |  |  |  |  |  |  | 1.0 |
| I50.0 | CHF | 3.9 | 3.6 | 4.6 | 3.3 | 4.5 | 2.1 | 5.5 | 3.4 |
| I63.5 | Cerebral infarction due to unspec. occlusion/stenosis cerebral arteries |  |  |  |  | 1.3 | 1.4 |  |  |
| I63.9 | Cerebral infarction, unspec |  |  |  | 0.9 |  |  |  |  |
| **[Total]** | | **[5.4]** | **[4.4]** | **[6.7]** | **[5.4]** | **[5.8]** | **[3.5]** | **[6.5]** | **[4.4]** |
| **J00-J99-Diseases of respiratory system** | |  |  |  |  |  |  |  |  |
|  |  | **DSL** | **LTC** | **DSL** | **LTC** | **DSL** | **LTC** | **DSL** | **LTC** |
| J10.1 | Influenza with other respiratory manifestations, seasonal inf virus identif |  |  |  |  |  |  | 1.5 | 1.0 |
| J18.9 | Pneumonia, unspec. | 1.9 | 1.6 | 5.1 | 3.9 | 3.7 | 1.0 | 5.2 | 6.0 |
| J44.0 | COPD with acute lower resp infection | 3.9 |  | 5.2 | 2.9 | 1.5 | 2.8 | 3.7 | 3.0 |
| J44.1 | COPD with acute exacerbation, unspec | 1.9 |  | 4.0 | 2.3 | 1.3 | 1.4 | 3.7 | 2.1 |
| J69.0 | Pneumonitis due to food & vomit (aspiration pneumonia) | 2.4 | 7.2 | 3.3 | 9.6 | 3.2 | 4.5 | 2.9 | 8.8 |
| J96.90 | Respiratory failure (hypoxic), unspec., not elsewhere classified |  |  |  | 1.2 |  |  |  |  |
| J96.91 | Respiratory failure (hypercapnic)-unspec, not elsewhere classified |  | 3.6 |  |  |  |  |  |  |
| **[Total]** | | **[10.1]** | **[12.4]** | **[17.6]** | **[19.9]** | **[9.7]** | **[9.7]** | **[17.0]** | **[20.9]** |
| **K00-K93-Diseases of digestive system** | |  |  |  |  |  |  |  |  |
| K56.5 | Intestinal adhesions with obstruction |  | 1.2 |  |  |  |  |  |  |
| K56.6 | Other & unspec. Intestinal obstruction |  |  |  |  | 1.0 | 1.7 |  | 1.0 |
| K57.3 | Diverticular disease of large intestine without perforation or abscess |  |  | 1.0 |  |  |  |  |  |
| K92.2 | Gastrointestinal haemorrhage, unspec. |  |  |  |  |  |  |  | 1.8 |
|  | **[Total]** |  | **[1.2]** | **[1.0]** |  | **[1.0]** | **[1.7]** |  | **[2.8]** |
| **L00-L99-Diseases of skin & subcutaneous tissue** | |  |  |  |  |  |  |  |  |
| L03.11 | Cellulitis of lower limb | 1.5 |  | 1.0 | 1.4 |  |  | 1.1 | 1.2 |
| **N00-N99-Diseases of genitourinary system** | |  |  |  |  |  |  |  |  |
| N17.9 | Acute renal failure, unspec | 1.9 | 2.0 | 1.3 |  |  | 1.4 | 1.1 |  |
| N39.0 | UTI, site not specified | 3.4 | 9.2 | 4.9 | 8.7 | 4.7 | 7.3 | 6.7 | 6.5 |
| **[Total]** | | **[5.3]** | **[11.2]** | **[6.2]** | **[8.7]** | **[4.7]** | **[8.7]** | **[7.8]** | **[6.5]** |
| **R00-R99-Symptoms, signs & abnormal clinical /lab findings not elsewhere classified** | |  |  |  |  |  |  |  |  |
| R41.80 | Transient alteration of awareness |  |  |  | 1.7 | 1.0 |  |  |  |
| R53 | Malaise & fatigue |  |  | 1.1 |  |  |  | 1.0 |  |
| R64 | Cachexia |  |  | 1.0 |  | 1.0 |  |  |  |
|  | **[Total]** |  |  | **[2.1]** | **[1.7]** | **[2.0]** |  | **[1.0]** |  |
| **S00-T98-Injury, poisoning & certain other consequences of external causes** | |  |  |  |  |  |  |  |  |
| S06.5 | Traumatic subdural haemorrhage |  |  |  | 0.9 |  |  |  |  |
| S32.500 | Fracture of pubis-closed | 1.5 |  |  |  | 1.3 |  | 1.2 |  |
| S72.080 | Fracture of neck of femur-closed | 4.4 | 4.4 | 2.5 | 2.1 | 3.0 | 2.8 | 1.8 | 2.2 |
| S72.090 | Fracture of neck of femur-closed | 1.9 |  |  | 0.9 | 2.0 | 1.0 | 1.2 | 1.1 |
| S72.100 | Intertrochanteric fracture-closed | 6.3 | 3.6 | 3.2 | 2.1 | 3.0 | 2.4 | 2.6 | 2.1 |
| T84.54 | Infection & inflammatory reaction due to knee prosthesis |  | 1.6 |  |  |  |  |  |  |
|  | **[Total]** | **[14.1]** | **[9.6]** | **[5.7]** | **[6.0]** | **[9.3]** | **[6.2]** | **[6.8]** | **[5.4]** |
| **Z00-Z99-Factors influencing health status & contact with health system** | |  |  |  |  |  |  |  |  |
| Z51.5 | Palliative care | 3.9 | 4.4 | 4.4 | 4.1 | 3.7 | 4.5 | 2.8 | 4.4 |
| **COVID-19** | |  |  |  |  |  |  |  |  |
| U07.1 | Confirmed with positive lab result / virus identified – lab confirmed |  | 4.8 |  |  | 15.0 | 11.1 |  |  |
| U07.2 | Clinical diagnosis / suspected clinically-probable / virus not identified |  |  |  |  |  |  |  |  |
