# Supplementary material for: Comparison of hospitalization events among residents of assisted living and nursing homes during COVID-19: Do settings respond differently during public health crises?
Source: PLoS One. 2024 Jul 12;19(7):e0306569. doi: 10.1371/journal.pone.0306569 (PMC11244779; doi:10.1371/journal.pone.0306569)
Supplement: S2 Fig — (DOCX) [file pone.0306569.s005.docx]

**S2 Fig. Monthly hospitalization with delayed discharge rate (per 100 person-days) across study period, January 1, 2018 to December 31, 2021, among Assisted Living (AL) and Nursing Home (NH) residents.**

Note: Red arrows: wave 1-4 monthly peaks; Yellow arrow: notable rise in hospitalization rate AL relative to NH; Grey arrow: month with low COVID-19 cases provincially & before vaccinations in settings; COVID-19 vaccinations started in settings January 1, 2021 and by May 28, 2021 ≥85% had received 2^nd^ dose.
