# Supplementary material for: Comparison of hospitalization events among residents of assisted living and nursing homes during COVID-19: Do settings respond differently during public health crises?
Source: PLoS One. 2024 Jul 12;19(7):e0306569. doi: 10.1371/journal.pone.0306569 (PMC11244779; doi:10.1371/journal.pone.0306569)

**S5 Fig. Monthly rate (per 100 residents) of positive COVID-19 tests, across pandemic period, March 1, 2020 to December 31, 2021, among Assisted Living (AL) and Nursing Home (NH) residents.**


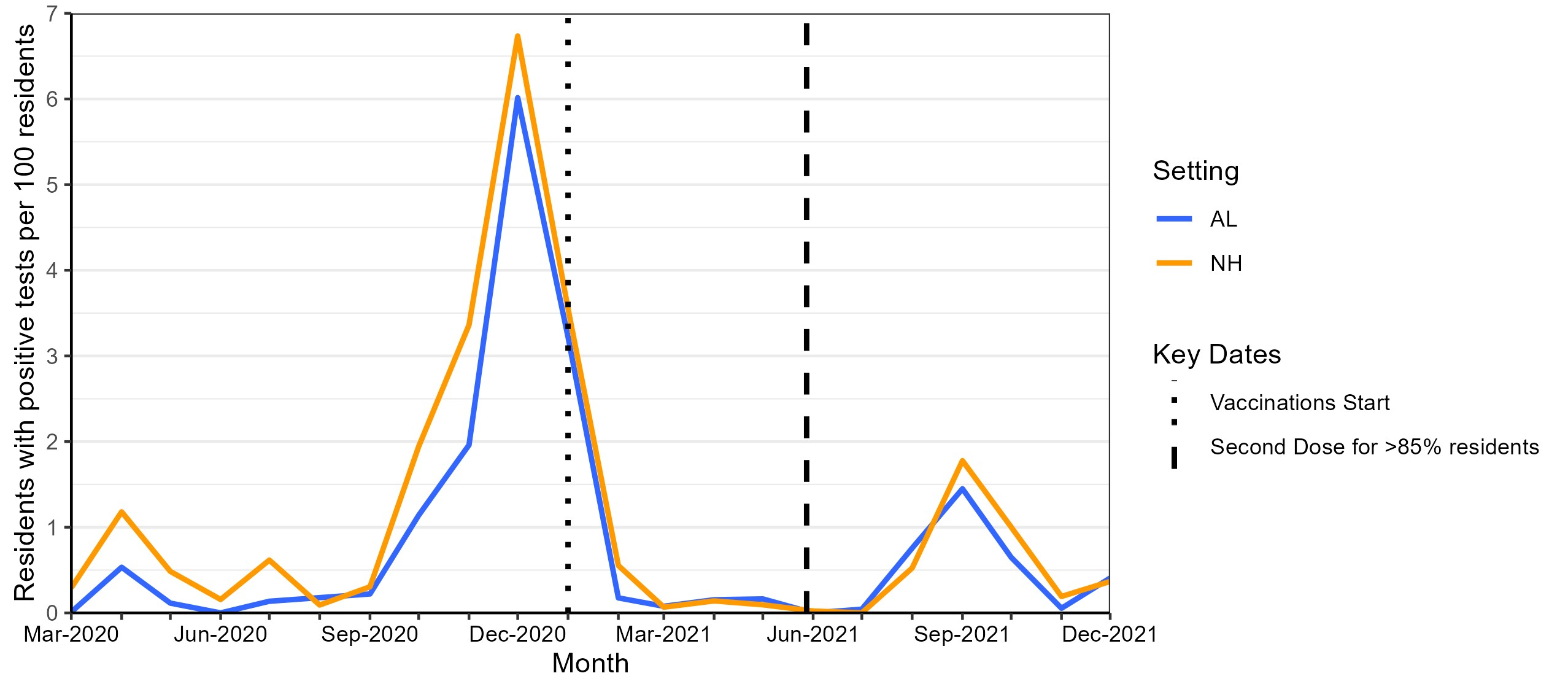

Supplement: S5 Fig — (DOCX) [file pone.0306569.s008.docx]
